# Antioxidant Potential of Herbal Preparations and Components from* Galactites elegans* (All.) Nyman ex Soldano

**DOI:** 10.1155/2018/9294358

**Published:** 2018-10-16

**Authors:** Omar Tebboub, Roberta Cotugno, Feyza Oke-Altuntas, Mohamed Bouheroum, Íbrahim Demirtas, Massimiliano D'Ambola, Nicola Malafronte, Antonio Vassallo

**Affiliations:** ^1^Unité de Recherche de Valorisation des Ressources Naturelles, Molécules Bioactives, Analyses Physico-Chimiques et Biologiques (VARENBIOMOL), Faculty of Exact Sciences, Université des Frères Mentouri Constantine 1, Algeria; ^2^Dipartimento di Farmacia, Università di Salerno, Fisciano, (SA), Italy; ^3^Department of Biology, Faculty of Science, Gazi University, Ankara 06500, Turkey; ^4^Laboratory of Plant Research, Department of Chemistry, Faculty of Science, Cankiri Karatekin University, Cankiri, Turkey; ^5^Dipartimento di Scienze, Università degli Studi della Basilicata, Potenza, Italy

## Abstract

*Galactites *is a genus of flowering plants belonging to Asteraceae family. This genus is mainly represented by the* Galactites elegans *(All.) Nyman ex Soldano, the milky thistle, a plant of Mediterranean origin.* Galactites elegans *is consumed as a monofloral boar thistle honey. Chromatography separation of CHCl_3_ and* n-*BuOH extracts of aerial parts of* G. elegans *led to isolation of 18 pure compounds. Their structures were elucidated by 1D-and 2D-NMR spectroscopy and confirmed by mass spectrometry analysis. Sinapic aldehyde, abietin, chlorogenic acid, neochlorogenic acid, 8*α*-hydroxypinoresinol, 9*α*-hydroxypinoresinol, pinoresinol, 4-ketopinoresinol, nortrachelogenin, and* erythro*-guaiacylglycerol-*β*-*O*-4′-dihydroconiferyl alcohol were isolated from CHCl_3_ extract, while luteolin 4′-*O*-glucuronide, naringenin*-*7-*O*-neohesperidoside, kaempferol-3-*O*-*α*-L-rhamnopyranosyl-(1→6)-*β*-D-glucopyranoside, apigenin-7-*O*-*α*-L-rhamnopyranosyl-(1→6)-*β*-D-glucopyranoside, quercitrin, quercetin-3-*O*-*α*-L-rhamnopyranosyl-(1→6)-*β*-D-glucopyranoside, ciwujiatone, and nortrachelogenin-4,4′-di-*O*-*β*-D-glucopyranoside were obtained from* n-*BuOH extract. The majority of isolated compounds displayed a significant antioxidant potential* in vitro *test (DPPH). The ability of compounds to reduce the level of peroxides in control and BHP-treated Jurkat cells was studied. The lignan derivatives were also able to reduce at 50 *μ*M the basal level of peroxides in Jurkat cells as well as counteract peroxide increase induced by BHP treatment. Particularly 8*α*-hydroxypinoresinol was the most active showing 70% of peroxide level inhibition.

## 1. Introduction

*Galactites *is a genus of flowering plants belonging to Asteraceae Compositae (commonly referred to as the aster, daisy, composite, or sunflower family) which is a very large and widespread family of flowering plants (Angiospermae). Many members belonging to this family are herbaceous, but a significant number are also shrubs, vines, or trees. The family has a worldwide distribution most commonly in the arid and semiarid regions of subtropical and lower temperate latitudes [1]. Asteraceae is an economically important family, providing products such as cooking oils, lettuce, sunflower seeds, artichokes, sweetening agents, coffee substitutes, and herbal teas. Plants in Asteraceae are medically important in areas that do not have access to Western medicine. They are also commonly featured in medical and phytochemical journals because the sesquiterpene lactone compounds contained within them are an important cause of allergic contact dermatitis [2].

This genus is mainly represented by the* Galactites elegans* (All.) Nyman ex Soldano, the milky thistle, a plant of Mediterranean origin (synonym:* Galactites tomentosa* Moench; common name: Scarlina).* Galactites elegans *is consumed as a monofloral boar thistle honey. This plant prefers sunny places and usually grows on the uncultivated or barren grounds, waste places, well-drained soils, pastures, and roadsides [3–5].

In our systematic search for polyphenolic constituents from Algerian plants, we have investigated the aerial parts of* Galactites elegans *and report herein isolation and structural elucidation of 18 compounds and their antioxidant activities.

## 2. Material and Methods

### 2.1. Chemicals and Reagents

Anhydrous sodium carbonate, Folin*-*Ciocalteu's phenol reagent, and methanol (analytical reagent and HPLC gradient grade) were purchased from Merck (Darmstadt, Germany). Ethylenediaminetetraacetic acid (EDTA), 2,2-diphenyl-1-picrylhydrazyl (DPPH), 3-(2-pyridyl)-5,6-bis(4-phenyl-sulphonic acid)-1,2,4-triazine (ferrozine), iron (II) chloride (FeCl_2_), gallic acid, 2,6-di-*tert*-butyl-4-methylphenol (BHT), butylated hydroxyanisole (BHA), and dimethylsulphoxide (DMSO) were purchased from Sigma-Aldrich GmbH (Taufkirchen, Germany). All other chemicals were analytical grade and obtained from either Sigma or Merck. RPMI-1640 medium was from BioWhittaker Lonza (NJ, USA). Fetal bovine serum (FBS) was from GIBCO (Life Technologies, Grand Island, NY, USA). 2′,7′-Dichlorofluorescein diacetate (DCFH-DA), tert-butyl hydroperoxide (BHP), and all the other chemicals were from Sigma-Aldrich (St. Louis, MO, USA)

### 2.2. General Experimental Procedures

Briefly optical rotations were measured on a Perkin*-*Elmer 241 polarimeter equipped with a sodium lamp (589 nm) and a 1 dm microcell. UV spectra were recorded on a Perkin*-*Elmer-Lambda spectrophotometer. NMR experiments were performed on a Bruker DRX-600 spectrometer at 300 K. HRESIMS were acquired in positive ion mode on a Q-TOF premier spectrometer equipped with a nanoelectrospray ion source (Waters-Milford, MA, USA). Column chromatography was performed over Sephadex LH-20 (Amersham Biosciences; Uppsala, Sweden). Silica gel 60 (0.040–0.063 mm; Carlo Erba; Milan, Italy) was used as column material. HPLC separation was conducted on a Shimadzu LC-8A series pumping system equipped with a Shimadzu RID-10A refractive index detector and Shimadzu injector on a C_18_*μ*-Bondapak column (30 cm x 7.8 mm, 10 *μ*m Waters, flow rate 2.0 mL min^−1^). TLC was performed on precoated Kiesel gel 60 F_254_ plates (Merck; Darmstadt, Germany); compounds were detected by Ce(SO_4_)_2_/H_2_SO_4_ (Sigma-Aldrich, Milan, Italy) solution; and reagent grade chemicals (Carlo Erba; Milan, Italy) were used throughout [6, 7].

### 2.3. Plant Material

The aerial parts of* Galactites elegans*, voucher specimen (Gae alg0312-2012), were collected in the end of March 2013 (flowering stage) in Hamma Bouziane, Constantine, Algeria. Fresh aerial parts were dried to constant weight at room temperature.

### 2.4. Extraction and Isolation

Dried and powdered aerial parts of* G. elegans *(966 g) were macerated with MeOH-H_2_O (8:2) at room temperature. The operation repeated 3 times. The hydromethanolic extract was concentrated to dryness (under low pressure). The residue was suspended in H_2_O and successively partitioned with petroleum ether for 1 time then CHCl_3_, EtOAc, and* n-*BuOH (3 mL ×300 mL, each), respectively, affording a CHCl_3_ soluble fraction (2 g), an EtOAc-soluble fraction (5.5 g), and a* n-*BuOH soluble fraction (19 g).

A part of butanolic extract (2.79 g) was submitted to chromatographic separation on a Sephadex LH-20 column, using MeOH as mobile phase; fractions were collected, analyzed by TLC on silica 60 F254 gel-coated glass sheets using CHCl_3_:MeOH:H_2_O (80:18:2, v/v/v) and* n-*BuOH–AcOH–H_2_O (60:15:25, v/v/v) as eluent, and grouped to obtain 26 fractions.

The compound luteolin 4′-*O*-glucuronide [8] (7.3 mg) was obtained directly from the fraction 22. Fraction 6 was chromatographed using RP18 HPLC with MeOH/H_2_O (42:58, v/v) as mobile phase (flow rate 2.0 mL min^−1^) to yield pure compound nortrachelogenin 4,4′-di-*O*-ß-D-glucopyranoside (**2**) [9] (1.1 mg, t_R_ 36 min). Fraction 8 was isolated using RP18 HPLC with MeOH/H_2_O (37:63, v/v) as mobile phase (flow rate 2.0 mL min^−1^) to yield pure compounds chlorogenic acid [10] (8.1 mg, t_R_ 8 min), neochlorogenic acid [11] (3.0 mg, t_R_ 9 min), naringenin-7-*O*-neohesperidoside [12] (19.2 mg, t_R_ 27 min), quercetin-3-*O*-*α*-L-rhamnopyranosyl-(1→6)-*β*-D-glucopyranoside [13] (3.0 mg, t_R_ 52 min), apigenin-7-*O*-*α*-L-rhamnopyranosyl-(1→6)-*β*-D-glucopyranoside [14] (1.4 mg, t_R_ 62 min), and Kaempferol-3-*O*-*α*-L-rhamnopyranosyl-(1→6)-*β*-D-glucopyranoside [13] (1.9 mg, t_R_ 82 min). Fraction 9 was separated using RP18 HPLC with MeOH/H_2_O (35:65, v/v) as mobile phase (flow rate 2.0 mL min^−1^) to yield pure compound quercitrin [15] (24.7 mg, t_R_ 23 min). Fraction 14 was chromatographed using RP18 HPLC with MeOH/H_2_O (2:3, v/v) as mobile phase (flow rate 2.0 mL min^−1^) to yield pure compound quercitrin [15] (5.7 mg, t_R_ 34 min).

A part of CHCl_3_ extract (1.87 g) was fractionated by column chromatography (CC) of Silica gel eluted with CHCl_3_ followed by increasing concentrations of MeOH in CHCl_3_ (between 1% and 100%), fractions were collected and monitored by TLC to obtain 20 fractions. Fraction 4 was chromatographed using RP18 HPLC with MeOH/H_2_O (2:3, v/v) as mobile phase (flow rate 2.0 mL min^−1^) to yield pure compounds ciwujiatone [16] (0.7 mg, t_R_ 10 min), 4-ketopinoresinol [17] (**3**) (1.9 mg, t_R_ 40 min), pinoresinol [18] (1.2 mg, t_R_ 48 min), and nortrachelogenin [19] (**1**) (2.1 mg, t_R_ 57 min). Fraction 10 was chromatographed using RP18 HPLC with MeOH/H_2_O (35:65, v/v) as mobile phase (flow rate 2.0 mL min^−1^) to yield pure compound 8*α*-hydroxypinoresinol [20] (**4**) (1.8 mg, t_R_ 10 min). The Fraction 11 was separated using RP18 HPLC with MeOH/H_2_O (2:3, v/v) as mobile phase (flow rate 2.0 mL min^−1^) to yield pure compound 9*α*-hydroxypinoresinol [21] G4 (2.2 mg, t_R_ 38 min). Fraction 12 was chromatographed using RP18 HPLC with MeOH/H_2_O (35:65, v/v) as mobile phase (flow rate 2.0 mL min^−1^) to yield pure compounds sinapic aldehyde [22] (0.7 mg, t_R_ 16 min) and abietin [23] (1.1 mg, t_R_ 17 min). Fraction 15 was chromatographed using RP18 HPLC with MeOH/H_2_O (7:18, v/v) as mobile phase (flow rate 2.0 mL min^−1^) to yield pure compound* erythro*-guaiacylglycerol-*β*-*O*-4′-dihydroconiferyl alcohol [24] (1.3 mg, t_R_ 64 min).

The structure of each compound was determined by NMR (see Figures S1-S11 in the Supplementary Material for the ^1^H NMR spectra of the tested lignans and glycosides).

### 2.5. Antioxidant Activity

#### 2.5.1. Determination of Total Phenolic Contents

Total phenolic contents of the samples were analyzed using the Folin-Ciocalteu reagent according to the method of Milella [25] using gallic acid as standard, with some modifications [26]. The fraction solutions were mixed with 0.2 mL of 50% Folin-Ciocalteu reagent and allowed to react for 3 min and 1 mL aqueous solution of 2% Na_2_CO_3_ was added. At the end of incubation for 45 min at room temperature, absorbance of each mixture was measured at 760 nm. The same procedure was also applied to the standard solutions of gallic acid. Total phenolic contents were expressed as *μ*g gallic acid equivalents per mg of the fractions.

### 2.6. DPPH Radical Scavenging Assay

Radical scavenging activity was determined by a spectrophotometric method based on the reduction of a methanol solution of DPPH using the method of Blois [27]. The sample solutions were added to 0.004% methanol solution of DPPH. The mixture was shaken vigorously and left to stand at room temperature for 30 min in the dark. The absorbance was measured at 517 nm against a blank by a spectrophotometer (Rayleigh, UV-2601). Scavenging of DPPH radical was calculated according to formula:

(1) $$\begin{array}{c} Radical\;\;scavenging\;\%=\left[\;\frac{\left(A_{control}-A_{sample}\right)}{A_{control}}\;\right]\times 100 \end{array}$$

where *A*_control_ is the absorbance of the control reaction (containing all reagents except the test compound) and *A*_sample_ is the absorbance of the test compound. DPPH scavenging activity was expressed as IC_50_ values (*μ*g/mL) for comparison. The IC_50_ value of each sample was defined as the concentration of sample required for a 50% decrease in absorbance of the blank. BHT and BHA were used as positive controls.

### 2.7. Metal Chelating Activity on Ferrous Ions (Fe^2+^)

Metal chelating activity was determined according to the method of Decker and Welch [28], with some modifications [29, 30]. Briefly, 0.5 mL of the samples was mixed with 0.05 mL 2 mM FeCl_2_ and 0.1 mL 5 mM ferrozine. The mixture was diluted with methanol (2 mL) and left standing at room temperature for 10 minutes. The absorbance of the solution was measured spectrophotometrically at 562 nm. EDTA was used as a positive control.

### 2.8. Cell Cultures

Jurkat cells (a T-*cell* leukemia cell line obtained from Cell Bank in GMP-IST, Genova, Italy) were maintained in RPMI 1640 medium supplemented with 10% (v/v) FBS, 2 mM L-glutamine, and antibiotics at 37°C in humidified atmosphere with 5% CO_2_. To ensure logarithmic growth, cells were subcultured every three days. Stock solutions (50 mM) of polyphenolic compounds in DMSO were stored at -20°C and appropriately diluted in the same solvent or directly in the medium just before use (DMSO never exceeding 0,5%).

### 2.9. Peroxide Depletion Activity of Test Compounds by Cytofluorometry

The evaluation of intracellular peroxides concentration was performed according to Rothe [30] with some modifications. In detail, Jurkat cells were collected by centrifugation and suspended in RPMI containing 5% FBS at a density of 5 x 10^5^ cells/mL. FBS concentration was lowered to 2% to increase the uptake rate of flavonoids and reagents in the short-incubation time (1 hr) chosen for the assay. Cell suspensions were incubated with increasing concentrations of each chemical or vehicle only at 37°C. For each sample duplicate test tubes were prepared. After 30 min of incubation, BHP (550 *μ*M final concentration) or an equal volume of vehicle was added. This allowed us to monitor the effect of flavonoids on BHP-induced peroxide elevation or the basal levels of peroxides, respectively. In the last 15 min of incubation cells were loaded with DCFH-DA (8 *μ*M final concentration). Test tubes were gentle mixed several times along the incubation period (1 hr). Cells suspension were then washed and resuspended in an equal volume of medium and 10,000 events were analyzed for DCF-fluorescence by cytofluorometry (BD FACSCalibur™ instrument, Becton Dickinson, San Jose, CA, USA). DCF green fluorescence was analyzed in the FL1 channel (*λ*_exc_ 488 nm; (*λ*_em_ 535 nm). Before the cytofluorometric analysis an aliquot of each sample was withdrawn to evaluate cell viability by Trypan-blue exclusion test.

### 2.10. Statistical Analysis

All experiments were performed in triplicate and the results were expressed as mean ± SD. Statistical analyses were performed using the SPSS 11.5 (SPSS, Chicago, IL). For DPPH activity, differences among means were done by analysis of variance (ANOVA), and averages were compared using the Duncan test. For other tests, differences between treatment groups were analyzed by the student test. Differences were considered significant when P <0.05.

## 3. Results and Discussion

### 3.1. Antioxidant Activities of Extracts and Components from* Galactites elegans*

#### 3.1.1. Total Phenolic Content

Phenolic compounds are characterized by having at least one aromatic ring with one or more hydroxyl groups attached which directly contribute to the antioxidant properties [31]. Therefore, it is important to evaluate the total phenolic in the extracts from* Galactites elegans*. The contents of total phenolic compounds in the extracts, expressed as *μ*g gallic acid equivalents per milligram of dry extract, are shown in Table 1.

#### 3.1.2. DPPH Radical Scavenging Activity

DPPH radical scavenging activities of the extracts and the reference synthetic agents are given in Table 1. According to these IC_50_ values, the DPPH radical scavenging abilities among the different extracts were in the order of CHCl_3_>* n-*BuOH (*P *< 0.05). Furthermore, pure compounds radical scavenging abilities is reported in Table 2. Lower IC_50_ value indicates higher free radical scavenging activity.

#### 3.1.3. Metal Chelating Activity

Iron ions catalyse the conversion of less reactive species such as H_2_O_2_ or lipid peroxides into more reactive ones such as hydroxyl or peroxyl/alkoxyl radicals. Therefore, extracts with iron chelating ability can act as powerful antioxidants [32]. The metal chelating ability of the extracts was investigated by ferrozine assay. The chelating potential of* n-*BuOH extract was determined as 38.5 ± 1.4 % that was significantly lower (*p*< 0.01) than synthetic chelating agent EDTA (93.7 ± 0.3 %) at the concentration of 2 mg/mL. On the other hand, chloroformic extract was not effective at the tested concentration.

### 3.2. Antioxidant Potential of Test Compounds by Cytofluorometry

**Figure 1:**
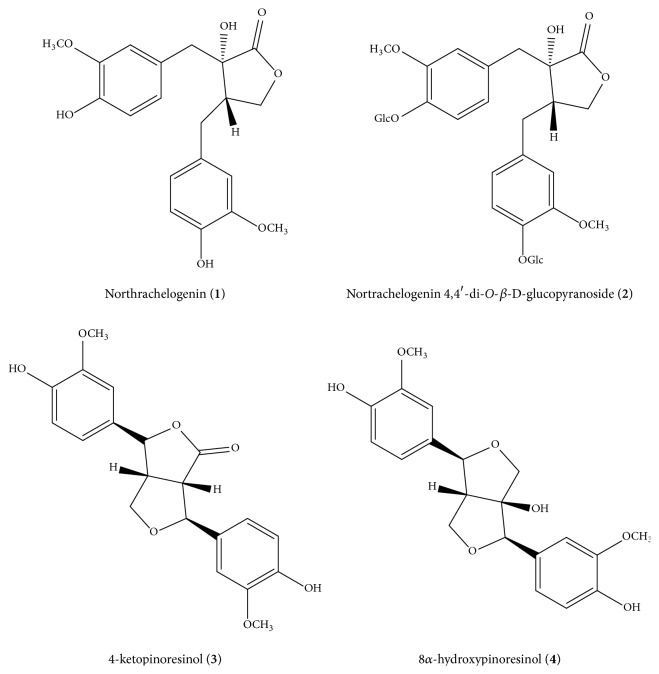
Structures of the tested lignans.

This study was carried out in order to discover natural compounds which could be used as active ingredients to improve the health and/or physical appearance of the skin or as preservatives or stabilizers for other active ingredients or vehicles in topical formulations. As reported in a recent work of Korte [33], natural products which show this kind of bioactivity are lignans and lignan esters. For these reasons, among all the tested molecules, pinoresinol and the northrachelogenin derivatives have been selected (Figure 1; see Figures S1-S4 in the Supplementary Material for the ^1^H NMR spectra of compounds).

Particularly, the* in cell *antioxidant potential of nortrachelogenin (**1**), nortrachelogenin-4,4′-di-*O*-*β*-D-glucopyranoside (**2**), and 8*α*-hydroxypinoresinol (**4**) have been investigated.

**Figure 2:**
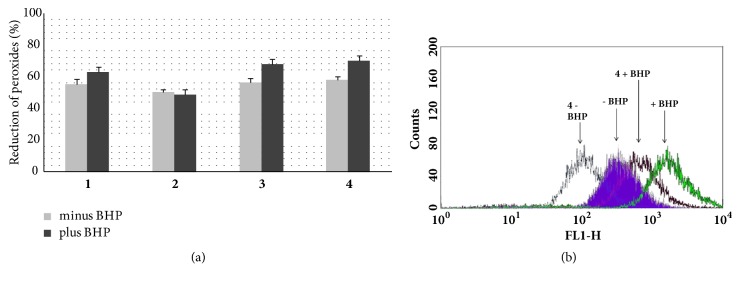
In-cells antioxidant potential of pinoresinol and the northrachelogenin derivatives. (a) Unstimulated (white bars) and BHP-stimulated (black bars) Jurkat cells were incubated with each tested compounds (50 *μ*M) or vehicle only. Cellular concentrations of peroxides (DCF fluorescence) were measured by cytofluorometry. Data shown were obtained using the mean fluorescence values and are the mean values ± SD of at least three experiments performed in duplicate. P values were always <0.01. (b) Representative histograms obtained with 8*α*-hydroxypinoresinol (**4**).

4-ketopinoresinol (**3**) was used as positive control as reported in Chen [17], however pinoresinol was not tested because of its high cytotoxicity, instead 9 hydropinoresinol was not active [21]. The ability of test compounds to reduce the level of peroxides in control and BHP-treated Jurkat cells was measured by cytofluorometry. Each chemical was tested at 25 *μ*M and 50 *μ*M concentrations, being the latter the maximum dose not cytotoxic. They were able to reduce the basal level of peroxides in* Jurkat cells* as well as counteract peroxide increase induced by BHP treatment. The slight lower antioxidant potential of nortrachelogenin 4,4′-di-*O*-ß-D-glucopyranoside (**2**) could be ascribed to the presence of a carbohydrate moiety which, if on one hand could slight contribute to the antioxidant activity and on the other hand might reduce nortrachelogenin 4,4′-di-O-ß-D-glucopyranoside availability for Jurkat cells. In addition, the sugar component seems to be responsible for the higher cytotoxic potential of nortrachelogenin 4,4′-di-*O*-ß-D-glucopyranoside, possibly due to perturbation of plasma membrane (Figure 2).

## 4. Conclusion

All the isolates (except chlorogenic acid) were first reported from the genus* Galactites *and the majority of isolated phenolic components displayed a significant antioxidant potential* in vitro* assay (DPPH). The lignan derivatives were also able to reduce at 50 *μ*M the basal level of peroxides in Jurkat cells as well as counteract peroxide increase induced by BHP treatment.

Particularly, 8*α*-hydroxypinoresinol (**4**) was the most active compound showing 70% of peroxide level inhibition. The slight lower antioxidant potential of nortrachelogenin 4,4′-di-*O*-*β*-D-glucopyranoside (**2**) (45% of peroxide level inhibition) could be ascribed to the presence of a carbohydrate moiety.

This result suggests that active fractions could be used as a source of antioxidant agent for pharmaceutical and cosmetic preparations.

## Figures and Tables

**Table 1:** Antioxidant activities of the extracts from *Galactites elegans*^1^.

Material	DPPH	Total phenolic content (*µ*gGAE/mg of material)	Metal chelating activity
	IC_50_ (*µ*g/mL)		(%)
Ex. CHCl_3_	41.2 ± 1.3^d^	116.5 ± 0.7^b^	NA^2^
Ex. *n-*Butanol	52.1 ± 1.1^c^	94.4 ± 0.6^a^	38.5 ± 1.4^b^
BHT	22.3 ± 0.8^b^	NS^3^	NS^3^
BHA	19.1 ± 0.4^a^	NS^3^	NS^3^
EDTA	NS^3^	NS^3^	93.7 ± 0.3^a^

^1^Values represent averages ± standard deviations for triplicate experiments. Values in the same column with different superscripts are significantly (*p *< 0.05) different. ^2^Not active. ^3^Not studied.

**Table 2:** IC_50_ values of polyphenols tested against the DPPH radical.

Compound	IC_50_ (*μ*M)^1^
BHT (positive control)	98.8 ± 4.5
BHA (positive control)	105.4 ± 5.3
4-ketopinoresinol (**3**)	143.3 ± 13.1
8*α*-hydroxypinoresinol (**4**)	71.5± 5.9
9*α*-hydroxypinoresinol	84.0± 2.9
Abietin	>500
apigenin-7-*O*-a-L-rhamnopyranosyl-(1→6)-*β*-D-glucopyranoside (isorhoifolin)	>500
chlorogenic acid	59.8 ± 4.9
Ciwujiatone	64.7 ± 5.3
*erythro*-guaiacylglycerol-*β*-*O*-4′-dihydroconiferyl alcohol	>500
kaempferol-3-*O*-*α*-L-rhamnopyranosyl-(1→6)-*β*-D-glucopyranoside (biorobin)	39.4 ± 2.8
luteolin 4′-*O*-glucuronide	38.9 ± 2.5
naringenin-7-*O*-neohesperidoside	116.0 ± 9.7
neochlorogenic acid	65.8 ± 4.8
nortrachelogenin (**1**)	38.6 ± 2.7
nortrachelogenin-4,4′-di-*O*-*β*-D-glucopyranoside (**2**)	34.4 ± 2.3
pinoresinol	50.8 ± 3.1
quercetin-3-*O*-*α*-l-rhamnopyranosyl-(1→6)-*β*-D-glucopyranoside (rutin)	63.0 ± 4.6
quercitrin	12.2 ± 1.0
sinapic aldehyde	53.4 ± 3.8

^1^Values represent averages ± standard deviations for triplicate experiments.

## Data Availability

The data used to support the findings of this study are available from the corresponding author upon request.

## Supplementary Materials

Supplementary Materials Figure S1: ^1^H NMR spectrum of compound** 1** (CD_3_OD, 600 MHz). Figure S2: ^1^H NMR spectrum of compound** 2** (CD_3_OD, 600 MHz). Figure S3: ^1^H NMR spectrum of compound** 3** (CD_3_OD, 600 MHz). Figure S4: ^1^H NMR spectrum of compound** 4** (CD_3_OD, 600 MHz). Figure S5: ^1^H NMR spectrum of abietin (CD_3_OD, 600 MHz). Figure S6: ^1^H NMR spectrum of luteolin 4′-*O*-glucuronide (CD_3_OD, 600 MHz). Figure S7: ^1^H NMR spectrum of naringenin-7-*O*-neohesperidoside (CD_3_OD, 600 MHz). Figure S8: ^1^H NMR spectrum of kaempferol-3-*O*-*α*-L-rhamnopyranosyl-(1→6)-*β*-D-glucopyranoside (CD_3_OD, 600 MHz). Figure S9: ^1^H NMR spectrum of apigenin-7-*O*-*α*-L-rhamnopyranosyl-(1→6)-*β*-D-glucopyranoside (CD_3_OD, 600 MHz). Figure S10: ^1^H NMR spectrum of quercitrin (CD_3_OD, 600 MHz). Figure S11: ^1^H NMR spectrum of quercetin-3-*O*-*α*-L-rhamnopyranosyl-(1→6)-*β*-D-glucopyranoside (CD_3_OD, 600 MHz).
