# Supplementary material for: Antioxidant Potential of Herbal Preparations and Components from Galactites elegans (All.) Nyman ex Soldano
Source: Evid Based Complement Alternat Med. 2018 Oct 16;2018:9294358. doi: 10.1155/2018/9294358 (PMC6206561; doi:10.1155/2018/9294358)
Supplement: Supplementary Materials — Figure S1: 1H NMR spectrum of compound 1 (CD3OD, 600 MHz). Figure S2: 1H NMR spectrum of compound 2 (CD3OD, 600 MHz). Figure S3: 1H NMR spectrum of compound 3 (CD3OD, 600 MHz). Figure S4: 1H NMR spectrum of compound 4 (CD3OD, 600 MHz). Figure S5: 1H NMR spectrum of abietin (CD3OD, 600 MHz). Figure S6: 1H NMR spectrum of luteolin 4′-O-glucuronide (CD3OD, 600 MHz). Figure S7: 1H NMR spectrum of naringenin-7-O-neohesperidoside (CD3OD, 600 MHz). Figure S8: 1H NMR spectrum of kaempferol-3-O-α-L-rhamnopyranosyl-(1→6)-β-D-glucopyranoside (CD3OD, 600 MHz). Figure S9: 1H NMR spectrum of apigenin-7-O-α-L-rhamnopyranosyl-(1→6)-β-D-glucopyranoside (CD3OD, 600 MHz). Figure S10: 1H NMR spectrum of quercitrin (CD3OD, 600 MHz). Figure S11: 1H NMR spectrum of quercetin-3-O-α-L-rhamnopyranosyl-(1→6)-β-D-glucopyranoside (CD3OD, 600 MHz). [file 9294358.f1.zip › 9294358.f1/9294358_SupplDesc.docx]

Figure S1. ^1^H NMR spectrum of compound **1** (CD_3_OD, 600 MHz).

Figure S2. ^1^H NMR spectrum of compound **2** (CD_3_OD, 600 MHz).

Figure S3. ^1^H NMR spectrum of compound **3** (CD_3_OD, 600 MHz).

Figure S4. ^1^H NMR spectrum of compound **4** (CD_3_OD, 600 MHz).

Figure S5. ^1^H NMR spectrum of abietin (CD_3_OD, 600 MHz).

Figure S6. ^1^H NMR spectrum of luteolin 4'-*O*-glucuronide (CD_3_OD, 600 MHz).

Figure S7. ^1^H NMR spectrum of naringenin-7-*O*-neohesperidoside (CD_3_OD, 600 MHz).

Figure S8. ^1^H NMR spectrum of kaempferol-3-*O*-α-L-rhamnopyranosyl-(1→6)-β-D-glucopyranoside (CD_3_OD, 600 MHz).

Figure S9. ^1^H NMR spectrum of apigenin-7-*O*-α-L-rhamnopyranosyl-(1→6)-β- D-glucopyranoside (CD_3_OD, 600 MHz).

Figure S10. ^1^H NMR spectrum of quercitrin (CD_3_OD, 600 MHz).

Figure S11. ^1^H NMR spectrum of quercetin-3-*O*-α-L-rhamnopyranosyl-(1→6)-β-D-glucopyranoside (CD_3_OD, 600 MHz).
