# Supplementary material for: Antioxidant Potential of Herbal Preparations and Components from Galactites elegans (All.) Nyman ex Soldano
Source: Evid Based Complement Alternat Med. 2018 Oct 16;2018:9294358. doi: 10.1155/2018/9294358 (PMC6206561; doi:10.1155/2018/9294358)

Supplementary Material

Antioxidant Potential of Herbal Preparations and Components from *Galactites elegans* (All.) Nyman ex Soldano.

Omar Tebboub,[1,§] Roberta Cotugno,[2,§] Feyza Oke-Altuntas,[3] Mohamed Bouheroum,[1] Íbrahim Demirtas,[4] Massimiliano D'Ambola,[2] Nicola Malafronte,[2] and Antonio Vassallo [5]*

[1] Unit de Recherche de Valorisation des Ressources Naturelles, Molécules Bioactives, Analyses Physico chimiques et Biologiques(VARENBIOMOL), Faculty of Exact Sciences, Universitè des Frers Mentouri Constantine1, Algeria
[2] Dipartimento di Farmacia, Università di Salerno, Fisciano, (SA), Italy.
[3] Department of Biology, Faculty of Science, Gazi University, Ankara 06500, Turkey
[4] Laboratory of Plant Research, Department of Chemistry, Faculty of Science, Cankiri Karatekin University, Cankiri, Turkey
[5] Dipartimento di Scienze, Università degli Studi della Basilicata, Potenza, (PZ), Italy

[§] Equal contributing authors

*Correspondence should be addressed to Antonio Vassallo; antonio.vassallo@unibas.it

Contents

Figure S1. ^{1}H NMR spectrum of compound **1** (CD_3OD, 600 MHz).

Figure S2. ^{1}H NMR spectrum of compound **2** (CD_3OD, 600 MHz).

Figure S3. ^{1}H NMR spectrum of compound **3** (CD_3OD, 600 MHz).

Figure S4. ^{1}H NMR spectrum of compound **4** (CD_3OD, 600 MHz).

Figure S5. ^{1}H NMR spectrum of abietin (CD_3OD, 600 MHz).

Figure S6. ^{1}H NMR spectrum of luteolin 4'-*O*-glucuronide (CD_3OD, 600 MHz).

Figure S7. ^{1}H NMR spectrum of naringenin-7-*O*-neohesperidoside (CD_3OD, 600 MHz).

Figure S8. ^{1}H NMR spectrum of kaempferol-3-*O*-α-L-rhamnopyranosyl-(1→6)-β-D-glucopyranoside (CD_3OD, 600 MHz).

Figure S9. ^{1}H NMR spectrum of apigenin-7-*O*-α-L-rhamnopyranosyl-(1→6)-β- D-glucopyranoside (CD_3OD, 600 MHz).

Figure S10. ^{1}H NMR spectrum of quercitrin (CD_3OD, 600 MHz).

Figure S11. ^{1}H NMR spectrum of quercetin-3-*O*-α-L-rhamnopyranosyl-(1→6)-β-D-glucopyranoside (CD_3OD, 600 MHz).

Figure S1. ^{1}H NMR spectrum of compound **1** (CD_3OD, 600 MHz).

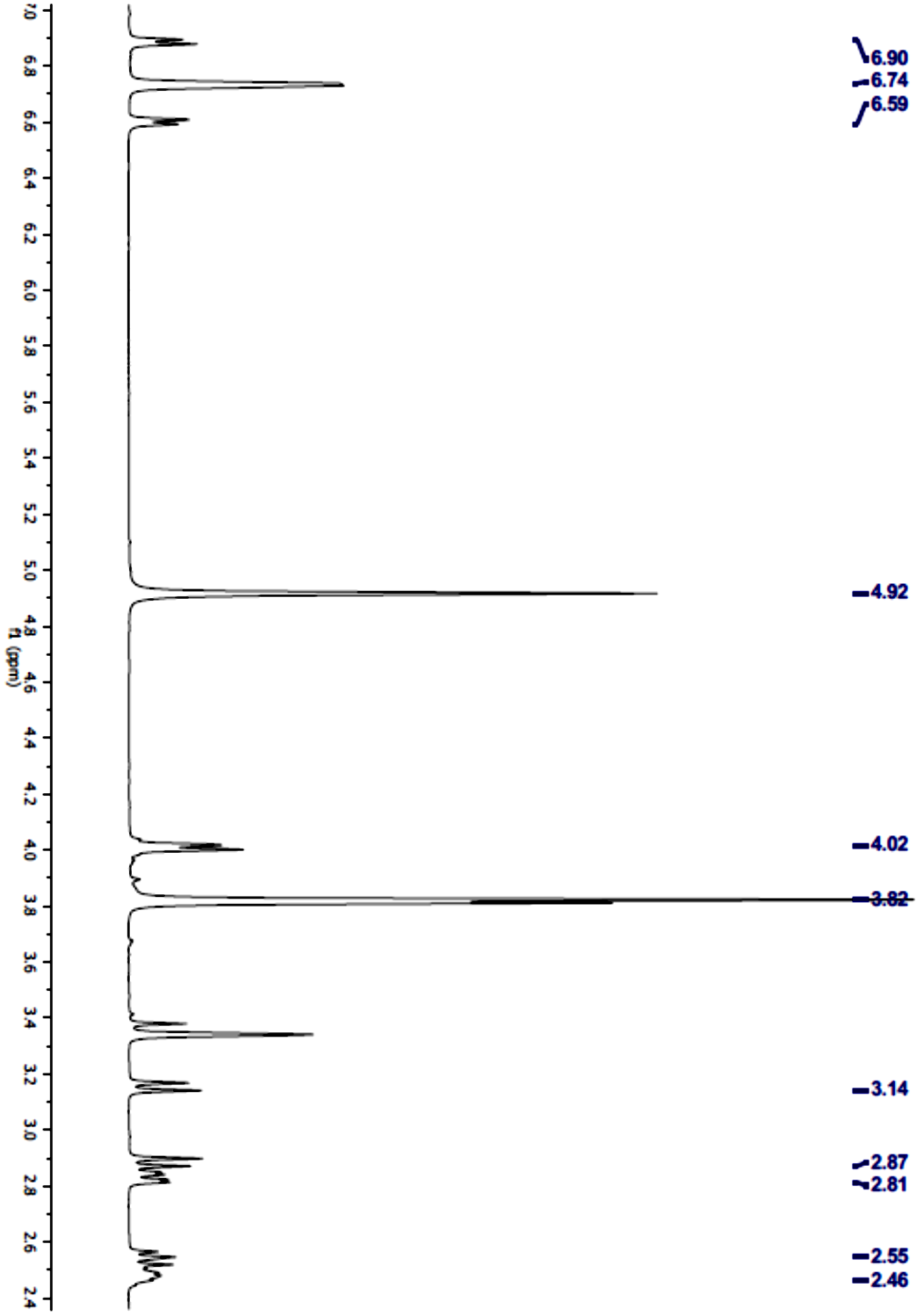

Figure S2. ^{1}H NMR spectrum of compound **2** (CD_3OD, 600 MHz).

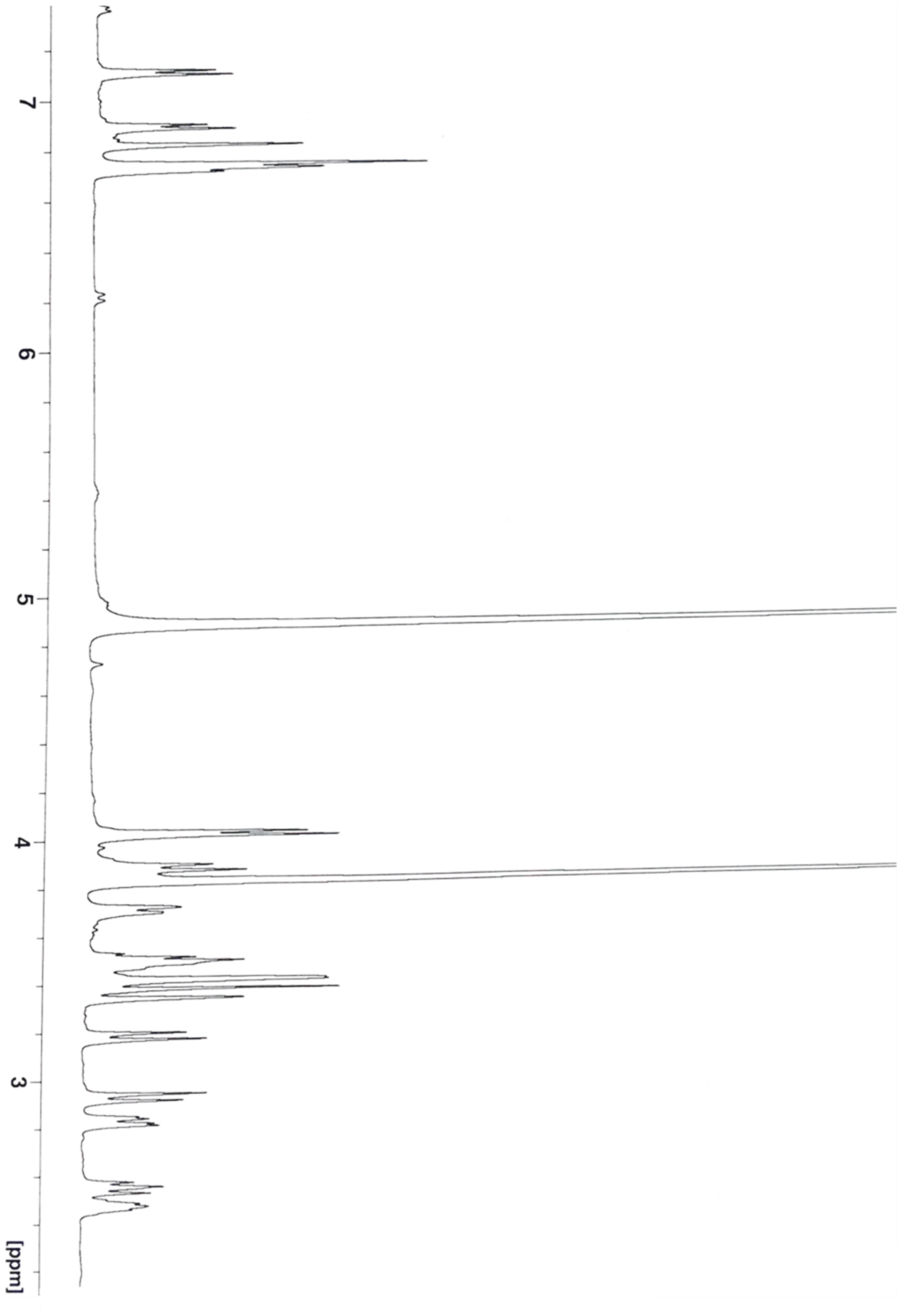

Figure S3. ^{1}H NMR spectrum of compound **3** (CD_3OD, 600 MHz).

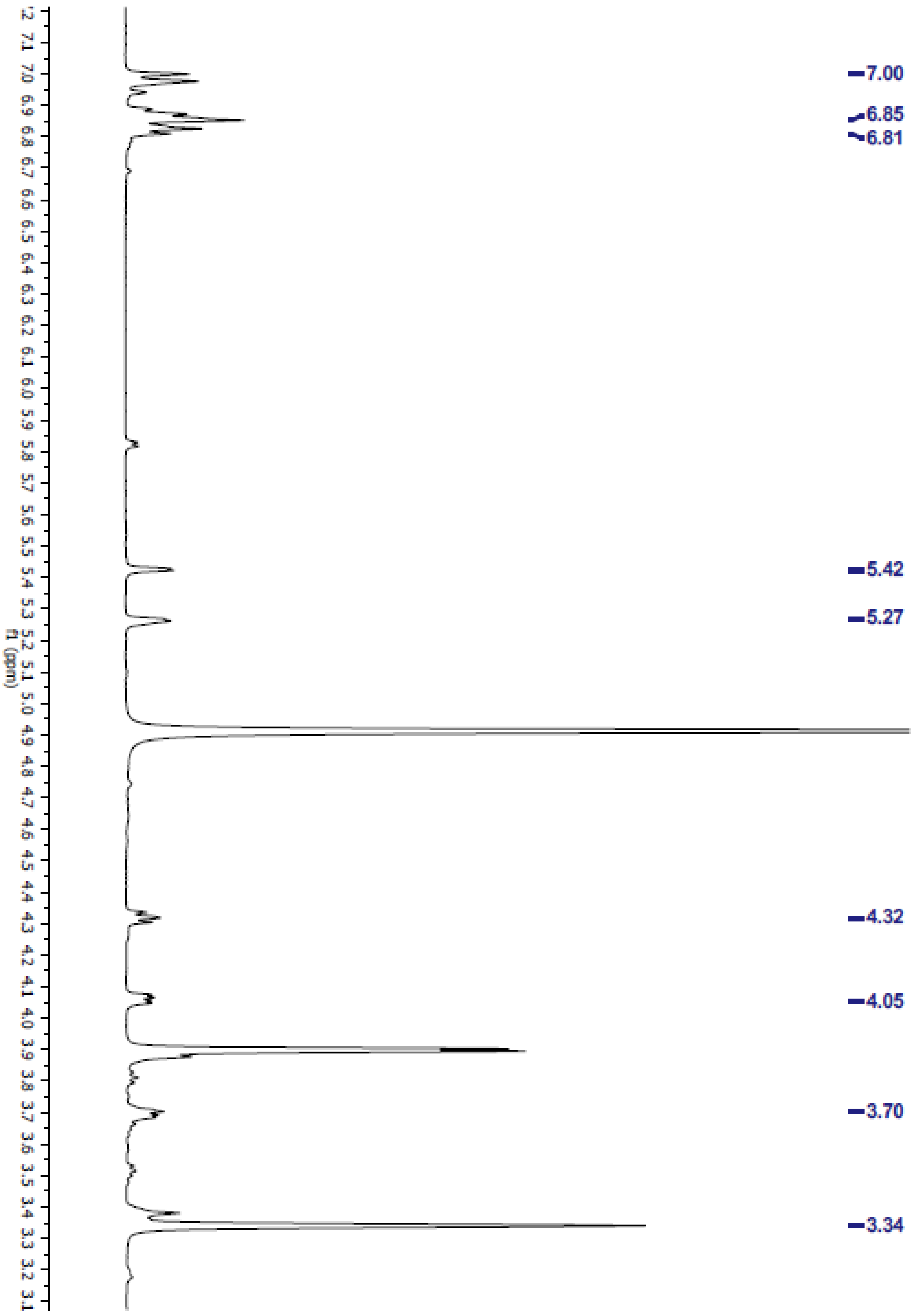

Figure S4. ^{1}H NMR spectrum of compound **4** (CD_3OD, 600 MHz).

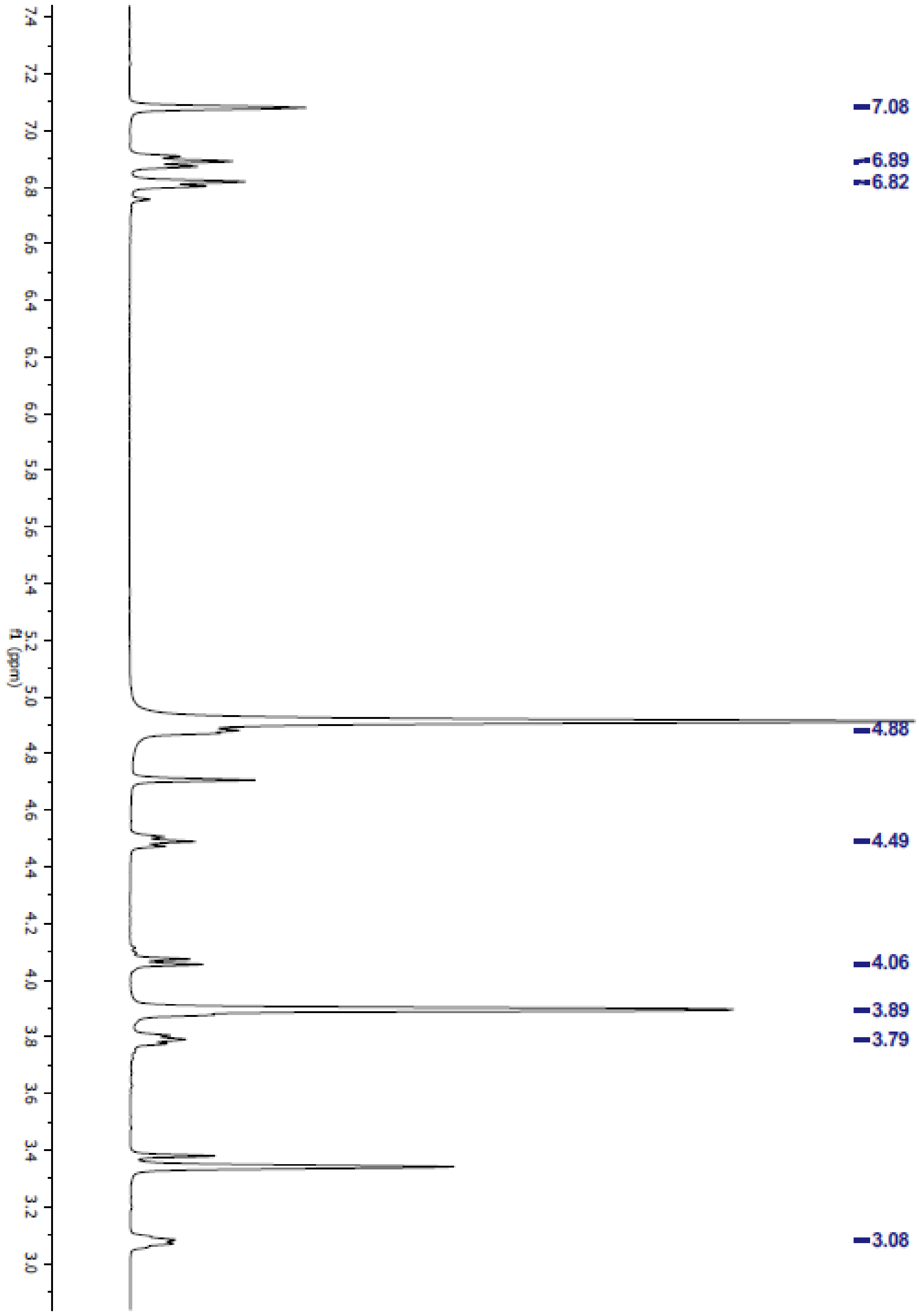

Figure S5. ^1H NMR spectrum of abietin (CD_3OD, 600 MHz).

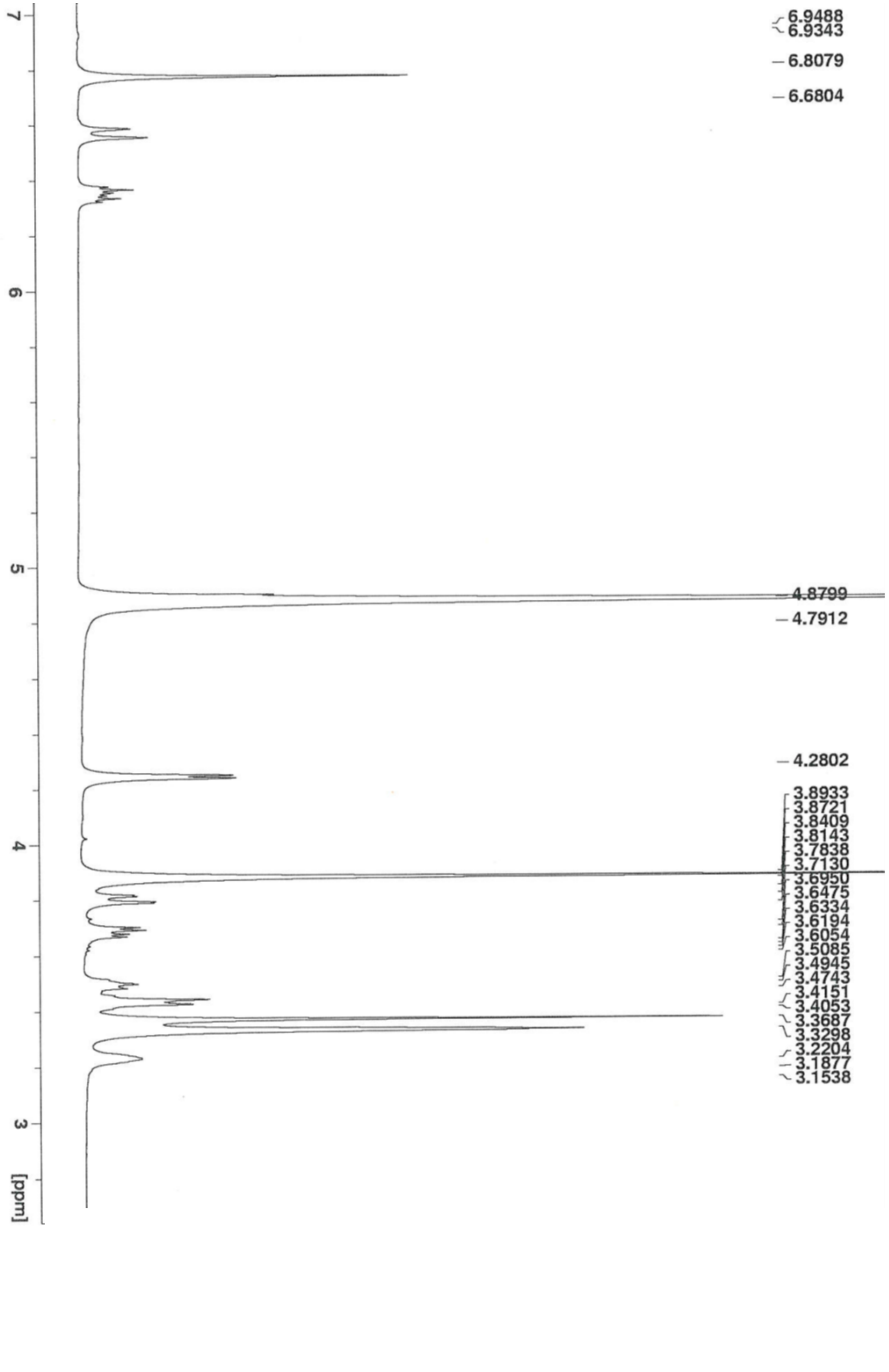

Figure S6. ^{1}H NMR spectrum of luteolin 4'-*O*-glucuronide (CD_3OD, 600 MHz).

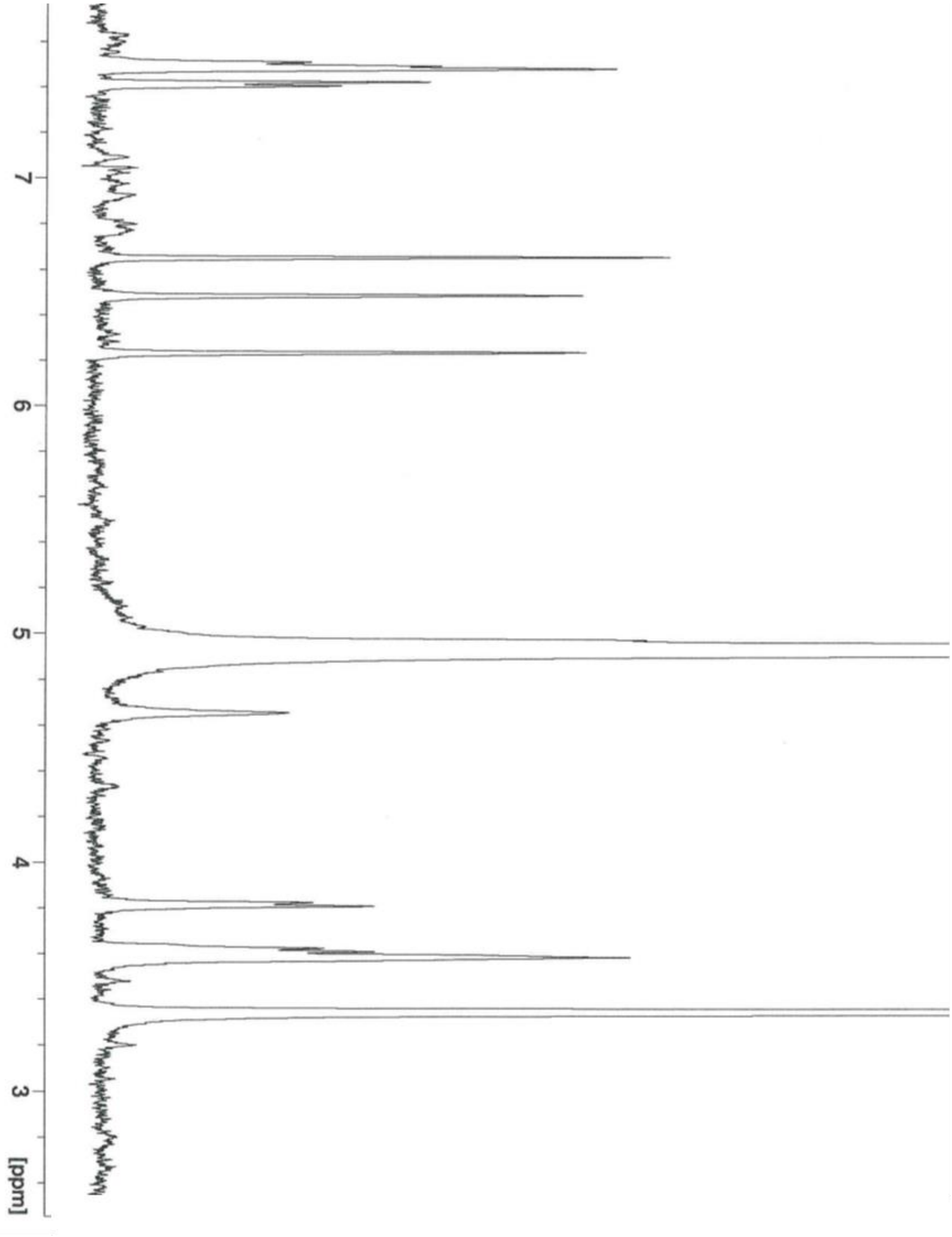

Figure S7. ^{1}H NMR spectrum of naringenin-7-*O*-neohesperidoside (CD_3OD, 600 MHz).

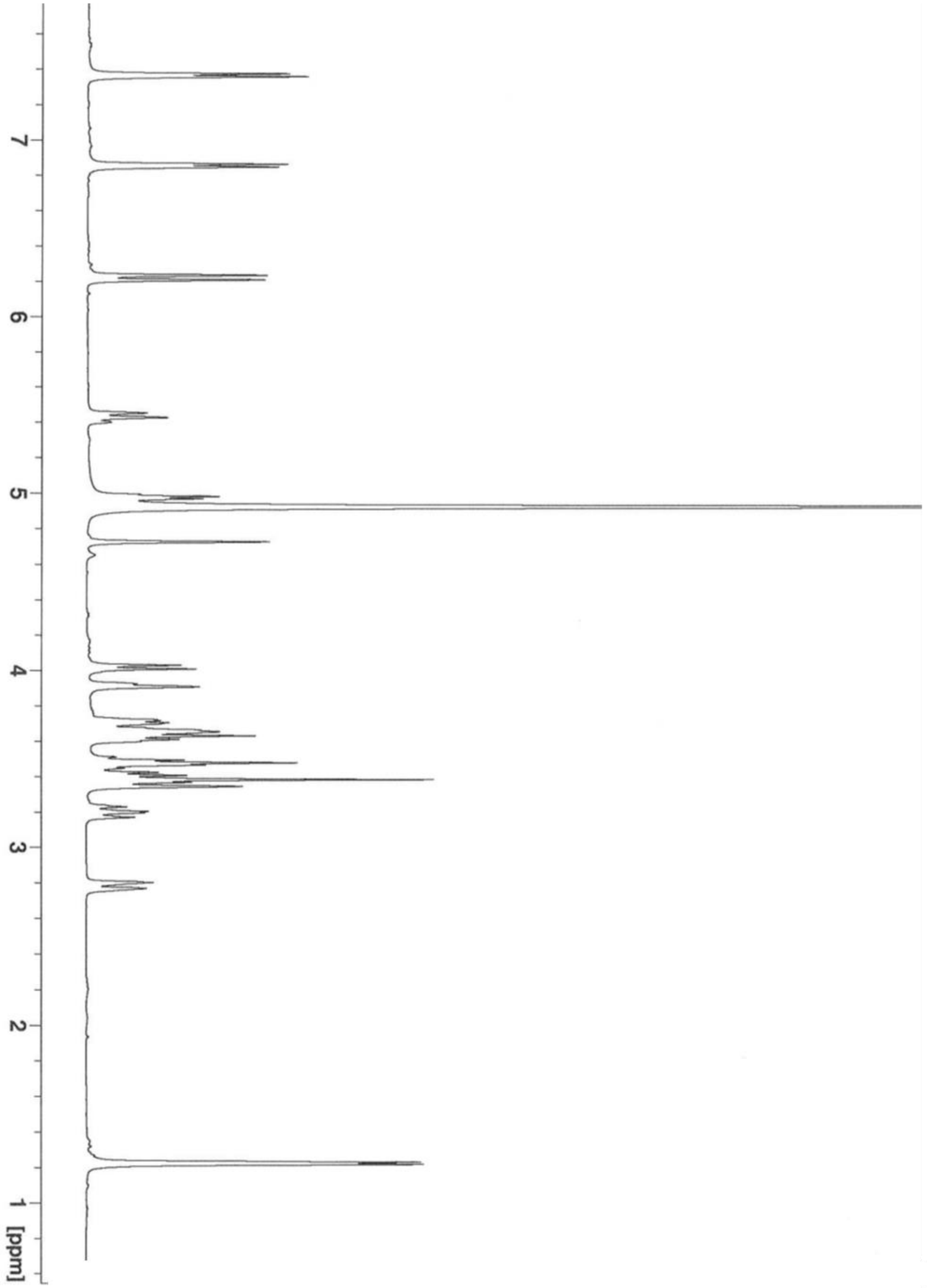

Figure S8. ^{1}H NMR spectrum of kaempferol-3-*O*-α-L-rhamnopyranosyl-(1→6)-β-D-glucopyranoside (CD_3OD, 600 MHz).

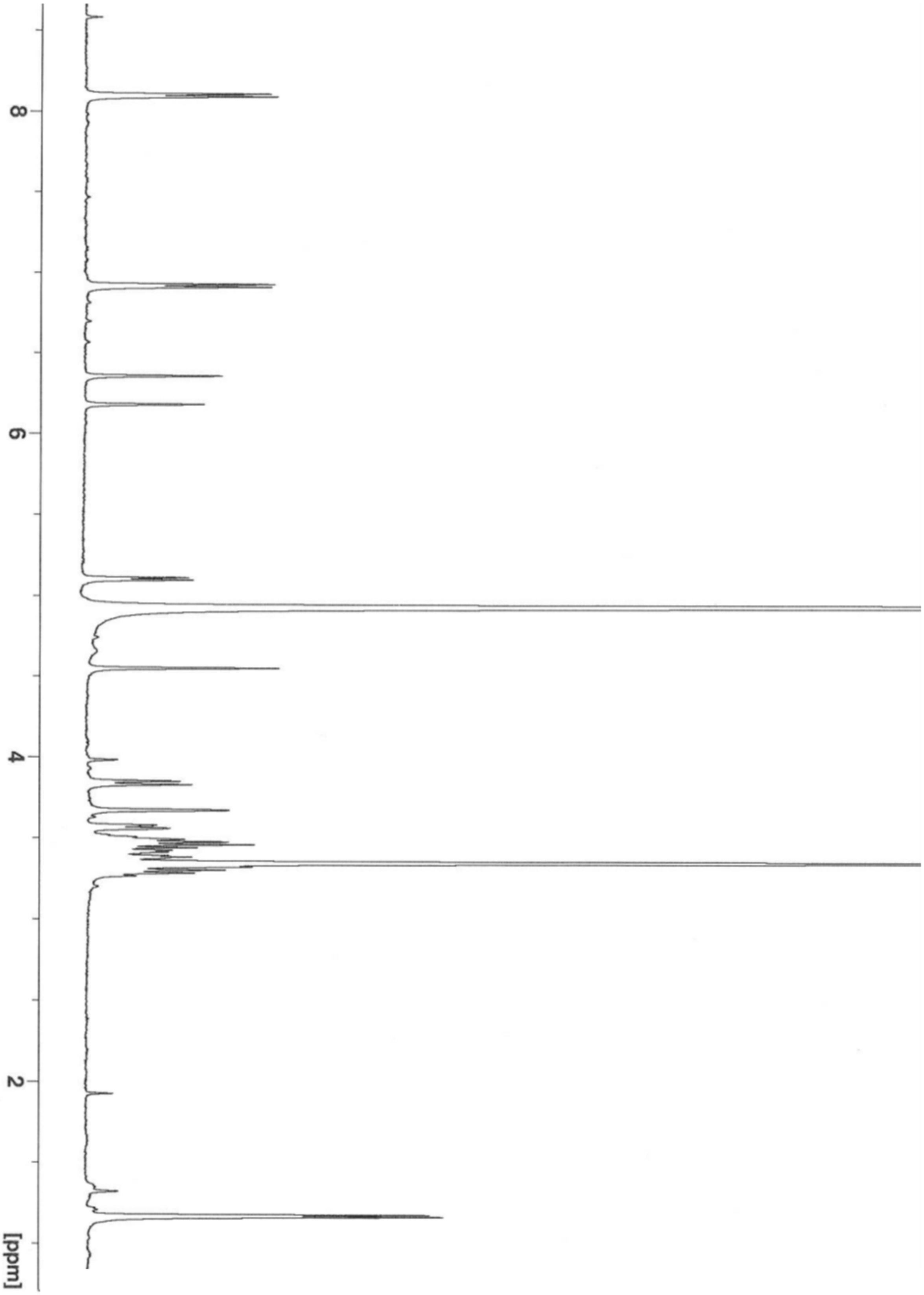

Figure S9. ^{1}H NMR spectrum of apigenin-7-*O*-α-L-rhamnopyranosyl-(1→6)-β- D-glucopyranoside (CD_3OD, 600 MHz).

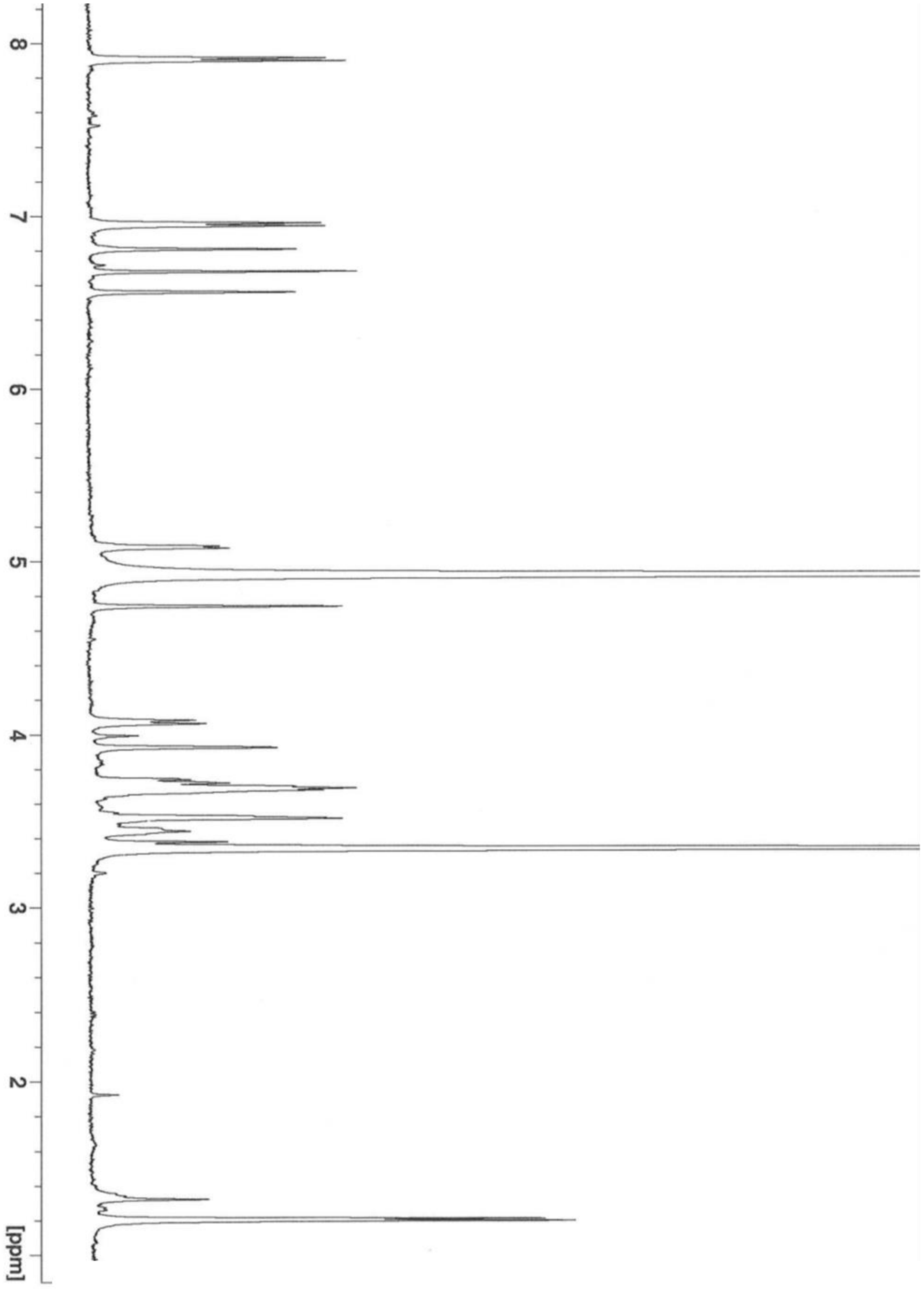

Figure S10. ^{1}H NMR spectrum of quercitrin (CD_3OD, 600 MHz).

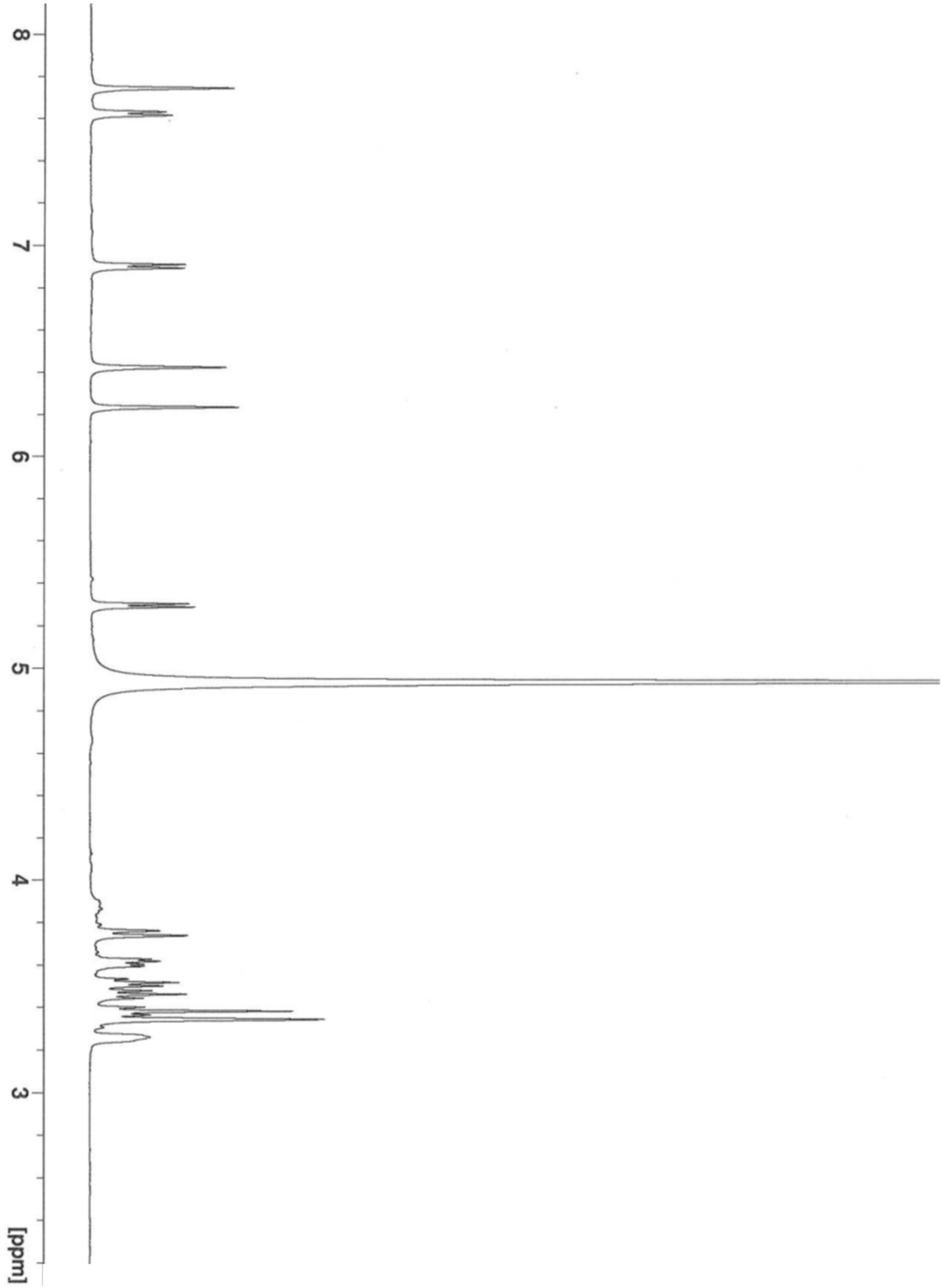

Figure S11. ^{1}H NMR spectrum of quercetin-3-*O*-α-L-rhamnopyranosyl-(1→6)-β-D-glucopyranoside (CD_3OD, 600 MHz).

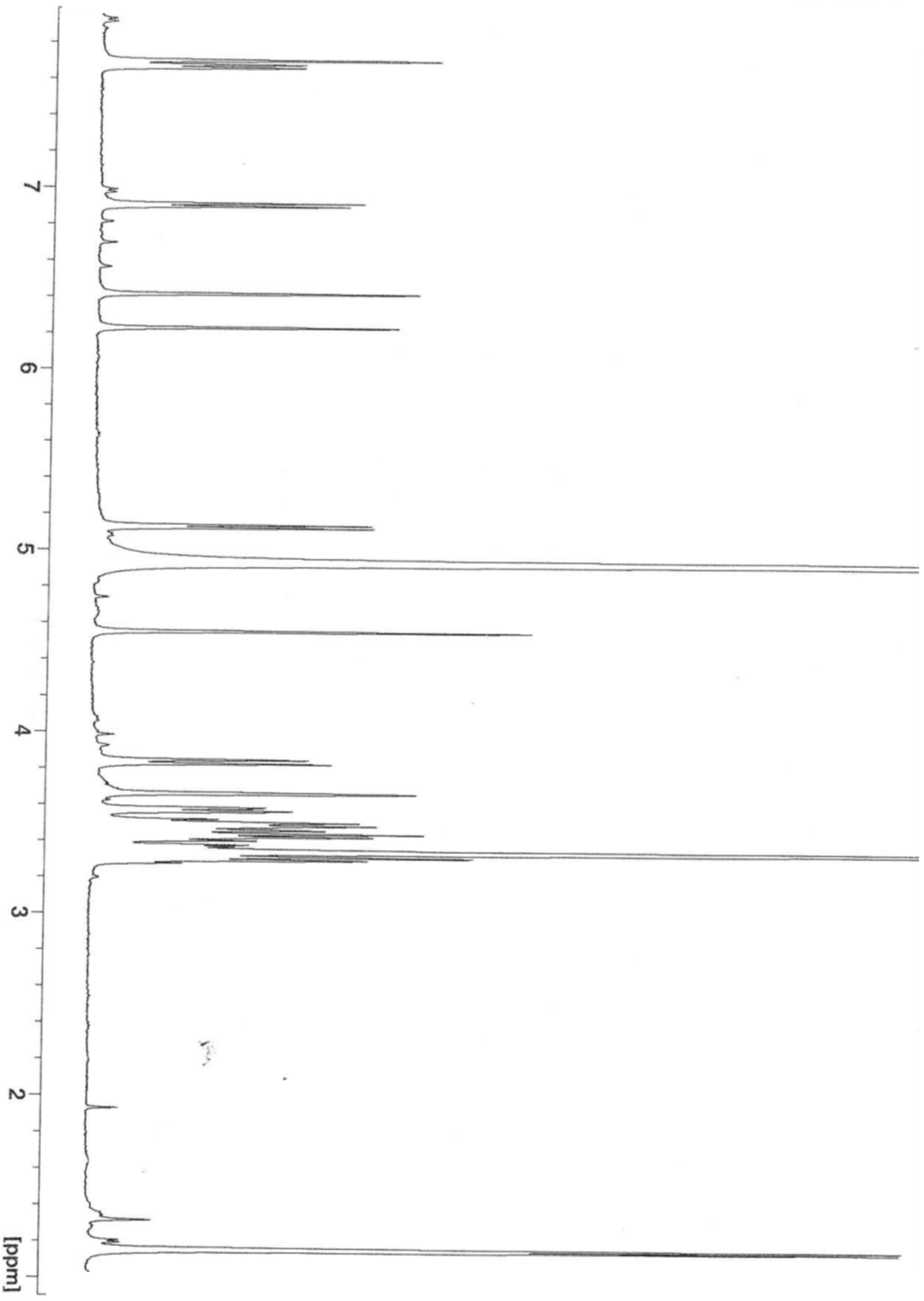

Supplement: Supplementary Materials — Figure S1: 1H NMR spectrum of compound 1 (CD3OD, 600 MHz). Figure S2: 1H NMR spectrum of compound 2 (CD3OD, 600 MHz). Figure S3: 1H NMR spectrum of compound 3 (CD3OD, 600 MHz). Figure S4: 1H NMR spectrum of compound 4 (CD3OD, 600 MHz). Figure S5: 1H NMR spectrum of abietin (CD3OD, 600 MHz). Figure S6: 1H NMR spectrum of luteolin 4′-O-glucuronide (CD3OD, 600 MHz). Figure S7: 1H NMR spectrum of naringenin-7-O-neohesperidoside (CD3OD, 600 MHz). Figure S8: 1H NMR spectrum of kaempferol-3-O-α-L-rhamnopyranosyl-(1→6)-β-D-glucopyranoside (CD3OD, 600 MHz). Figure S9: 1H NMR spectrum of apigenin-7-O-α-L-rhamnopyranosyl-(1→6)-β-D-glucopyranoside (CD3OD, 600 MHz). Figure S10: 1H NMR spectrum of quercitrin (CD3OD, 600 MHz). Figure S11: 1H NMR spectrum of quercetin-3-O-α-L-rhamnopyranosyl-(1→6)-β-D-glucopyranoside (CD3OD, 600 MHz). [file 9294358.f1.zip › 9294358.f1/mat.9294358.v3.pdf]
